# Fermentation Performance Evaluation of Lactic Acid Bacteria Strains for Sichuan Radish Paocai Production

**DOI:** 10.3390/foods13121813

**Published:** 2024-06-08

**Authors:** Yiwen Fan, Xu Yang, Cihai Hu, Banghong Wei, Fei Xu, Quanyou Guo

**Affiliations:** 1 School of Healthy Science and Engineering, University of Shanghai for Science and Technology, 516 Jungong Road, Shanghai 200093, China; 15737926563@163.com (Y.F.); 15637446157@163.com (C.H.); 2 Key Laboratory of Oceanic and Polar Fisheries, Ministry of Agriculture and Rural Affairs, East China Sea Fisheries Research Institute, Chinese Academy of Fishery Sciences, 300 Jungong Road, Shanghai 200093, China; yangxu@ecsf.ac.cn (X.Y.); weibanghong@ecsf.ac.cn (B.W.); 3 Shanghai Engineering Research Center of Food Rapid Detection, Shanghai 200093, China

**Keywords:** lactic acid bacteria, radish paocai, inoculated fermentation, flavor, TOPSIS

## Abstract

Fermented vegetable products play a significant role in various cuisines, and understanding the fermentation dynamics of lactic acid bacteria (LAB) strains is essential for optimizing their production and quality. Here, we sought to investigate the fermentation performance of five LAB strains isolated from Sichuan paocai as starters for paocai. Sensory evaluation revealed that the inoculation of radish paocai samples with LAB strains effectively improved the overall liking and sensory satisfaction of participants, increasing the scores to varying degrees in terms of taste, flavor, texture, and coloration. *Lactiplantibacillus plantarum* and *Lacticaseibacillus rhamnosus* exhibited a good salt resistance in radish juice and could grow in a medium containing 10% NaCl. Four indicator strains commonly found in contaminated paocai were effectively inhibited by fermented LAB broths, which improved the edibility and safe production of paocai. Compared to spontaneous fermentation (CK), radish paocai inoculated with LAB showed a significantly accelerated acid production rate, shortening the fermentation period by approximately two days. The contents of titratable total acids, organic acids, and free amino acids were higher in the inoculated samples and were enriched in the taste of radish paocai. The content of volatile organic compounds in the inoculated samples was higher than that in CK. Based on OPLS-DA analysis, 31 key indicators of paocai quality were screened and used to rank the fermentation performances of the five strains using the TOPSIS method; here, *Lpb. plantarum* and *Lcb. rhamnosus* achieved the highest scores. This study provides a reference for selecting LAB strains as efficient and secure fermentation starters to optimize paocai quality.

## 1. Introduction

Paocai is a generic term referring to fermented vegetable products [1]. Fermentation not only changes the original taste of the vegetables, but also improves their safety and storability [2,3]. Of the different types of paocai in China, Sichuan paocai is highly favored by consumers thanks to its unique flavor profile (sour and spicy tastes), crispy texture, and ease of consumption [4]. In particular, radish possesses nutritional value and palatability, making it one of the most important vegetables of Sichuan paocai [5]. Traditional Sichuan radish paocai is manufactured through spontaneous fermentation, which utilizes the native microbiota present in the raw ingredients and the environment. Therefore, the composition and load of the microbial community in the fermentation process may fluctuate due to variations in physicochemical and nutritional parameters and may be susceptible to contamination by undesirable microorganisms, resulting in potential food safety hazards, or inconsistent fermentation quality [6]. Currently, the fermentation environment of paocai in the industry is standardized by employing starter cultures for inoculated fermentation; this accelerates fermentation, while guaranteeing consistent fermentation kinetics [7]. Concomitantly, it enables the prediction and tailoring of food aroma profiles [8].

Bacteria exhibit higher diversity than fungi in the fermentative transformation of vegetables [9]. LAB strains constitute the predominant bacterial taxa that play a role in vegetable fermentation, especially *Weissella* sp., *Leuconostoc* spp., and *Lactobacillus* [10,11]. These are the dominant strains in numerous types of pickles such as zha-chili, Jiangxi yancai, and Dongbei suancai [12,13]. LAB strains can metabolize carbohydrates, proteins, and vitamins in vegetables to generate acids, alcohols, and FAAs, comprising an important flavor source in paocai [14]. LAB strains are widely distributed in nature, including in the majority of plants and even in soil, and there are also indigenous strains found in many naturally fermented foods. In addition, LAB strains are beneficial microbes in the human gastrointestinal tract, where they exhibit probiotic properties, highlighting the safety of LAB strains as starter cultures for fermented foods [15]. Current research on paocai has demonstrated that inoculation with LAB strains can enhance the flavor and textural attributes of paocai, substantially elevating their overall quality [1]. LAB strains can effectively suppress pathogenic microbes by synthesizing bacteriocins and antimicrobial peptides during fermentation, to improve the microbiological safety of the fermented products [16]. Differences may exist in the microbial community composition at different fermentation stages of paocai. Most paocai harbor abundant populations of *Leuconostoc mesenteroides*, *Lactobacillus lactis*, and *Weissella* spp. during the initial phase of fermentation. In contrast, *Lactobacillus brevis* exhibits a declining trend in relative abundance in the mid and late stages, whereas *Lpb. plantarum* persists throughout the fermentation process [6].

Given the ubiquitous presence of *Lpb. plantarum* in paocai, most studies have focused on this species and have obtained promising results. For example, *Lpb. plantarum* inoculation enhances the quality attributes of pickled peppers and maintains the microbiological safety of fermented carrots [17]. In addition, *Lpb. plantarum* can accelerate the fermentative kinetics of Dongbei Suancai and can mitigate nitrite accumulation in paocai [11,18]. Some studies have examined other LAB species, including *Lcn. mesenteroides* and *Lactococcus lactis* [11]. However, few studies have utilized *Lcb. Rhamnosus*, *Lcc. lactis*, or *Lvb. Brevis* as starter cultures, which could serve as potential starters for paocai in assessing its fermentative performance.

In a previous study, we isolated and identified five LAB species from traditional fermented Sichuan paocai, including *Lpb. plantarum*, *Lcb. rhamnosus, Pediococcus acidilactici*, *Lvb. Brevis*, and *Lentilactobacillus buchneri*. These strains are present in traditionally fermented paocai produced by local methods in Sichuan, exhibiting a good flavor and a consistent quality. The microbiological analysis of paocai samples indicates that *Lactobacillus* and *Leuconostoc* are the dominant microbial genera in the samples. The strains used in the experiment were isolated and selected from kimchi samples. They underwent physiological and biochemical tests, as well as preliminary evaluations of growth characteristics, acid production ability, nitrite reduction capacity, and fermentation performance. It is believed that these five lactic acid bacteria strains have an excellent potential for fermenting paocai [19]. Studies have demonstrated that *Weissella* is the predominant microorganism present during the initial and middle stages of fermentation [20]. Therefore, *Weissella paramesenteroides* obtained from the China Center of Industrial Culture Collection (CICC) was used as a control strain to evaluate the fermentative capacity of the five isolated LAB species. This study measured the physicochemical properties and flavor quality attributes of radish paocai in step 1, screened the critical quality indicators in step 2, and conducted sensory evaluation, correlation analysis, and comprehensive quality assessment in steps 3, 4, and 5. Using this systematic approach, an evaluation framework can be established for fermentative strains that are utilized as starter cultures in fermented foods. This study provides a theoretical basis and novel insights for the screening and characterization of potential starter cultures. 

## 2. Materials and Methods

### 2.1. Materials and Chemicals

Raw materials, including radish (*Raphanus sativus* L.), garlic (*Allium sativum* L.), ginger (*Zingiber officinale*), chili pepper (*Capsicum frutescens* L.), anise (*Pimpinella anisum*), myrcia (*Myrcia glomerata*), cassia (*Cinnamomum cassia*), Chinese prickly ash (*Zanthoxylum bungeanum Maxim*), and other seasoning ingredients, such as salts and sugars, were procured from a local supermarket in Shanghai, China. Organic acid standards were obtained from Dr. Ehrenstorger (Augsburg, Germany), while amino acid standards were obtained from Sigma-Aldrich (St. Louis, MO, USA). Culture media, including MRS, LB, YPD, and PDA, were acquired from Solarbio (Beijing, China). Chromatography-grade methanol and acetonitrile were used. Analytical-grade NaCl, sucrose, HCl, and NaOH were purchased from Sinopharm Chemical Reagent Co., Ltd. (Shanghai, China).

### 2.2. Source of LAB and Indicator Strains

The LAB bacteria used in this study, which include Lactiplantibacillus plantarum (PLP), Lacticaseibacillus rhamnosus (PLR), Pediococcus acidilactici (PPA), Levilactobacillus brevis (PLBR), and Lentilactobacillus buchneri (PLBU), were screened from traditional spontaneous Sichuan paocai and were identified by morphological and 16s rDNA gene sequencing analyses. As a reference strain, the standard Weissella paramesenteroides (CIW) strain with accession number CICC 24392 was obtained from CICC. The indicator microorganisms used in the antibacterial assays, including Escherichia coli, Bacillus subtilis, Saccharomyces cerevisiae, and Aspergillus niger were previously isolated from food spoilage samples. 

### 2.3. Determination of LAB Strain Growth Characteristics

#### 2.3.1. Gompertz Model Fitting of LAB Strain Growth Rates

Radish homogenate (200 g) was mixed with 9 g of sucrose and 200 mL of ultrapure water (UPW), cleared of impurities, and sterilized to obtain a radish juice culture medium. The experimental and reference LAB strains were inoculated into MRS broth and incubated at 37 °C for 24 h for activation. The activated bacterial cultures were adjusted to an OD_600_ of 1.00, before inoculating into the radish juice medium at a 10% inoculum ratio. The optical density (OD_600_) of the inoculum was monitored every 4 h using a Bioscreen (Bioscreen C, Turku, Finland). The Gompertz model, a bi-exponential function that describes asymmetric S-shaped microbial growth curves, was used to calculate the relevant growth parameters, including the maximum specific growth rate (MSGR) and the lag phase (LP) [21]. Gompertz model fitting was performed using Equation (1).

(1) $$N_{t}=N_{0}+(N_{\max}-N_{0})\times \exp\left\{-\exp\left[2.718\times \mu_{\max}\times \frac{\left(L\mathrm{ag}-t\right)}{\left(N_{\max}{-N}_{0}\right)}\right]\right\}$$

#### 2.3.2. Dynamic Monitoring of the pH and TTA of the Fermentation Liquid

The pH was determined using a pH meter (PHSJ-3F; Leici, Shanghai, China). Total titratable acidity (TTA) was analyzed following the Chinese National Standards GB 5009.239-2016 [22], using a digital burette (Brand, Titrette, Germany) after diluting the samples 10 times. All measurements were performed in triplicate.

#### 2.3.3. NaCl Tolerance of the LAB Strains

To analyze the growth profiles of the LAB strains under different NaCl concentrations, NaCl was added to the radish juice medium at 2%, 4%, 6%, 8%, 10%, and 12% (*w/v*). The measurement methods and procedures were identical to those described in Section 2.3.1.

#### 2.3.4. MIC Determination of Fermentation Supernatant

The antibacterial activities of the LAB strain fermentation supernatants were evaluated using the 96-well plate method described by Le et al. [23], with slight modifications. Briefly, the supernatants were obtained by inoculating the LAB stains into radish juice medium and incubating for 72 h, followed by centrifugation at 4 °C, for 10 min, at a 12,000× g force to remove cell debris. Next, 100 μL of indicator bacteria culture and 100 μL of supernatant were added to the first well of the 96-well plate. Serial dilutions (up to 10-fold) of the culture medium and cell-free supernatant were then performed in duplicate in the 96-well microplate, with the 11th and 12th columns serving as the positive and negative controls, respectively. This procedure was repeated three times for each sample, in triplicate. Bacterial suspensions of *E. coli*, *B. subtilis*, and *S. cerevisiae* were standardized to an OD_600_ of 1.00, while the spore suspension of *A. niger* was adjusted to 10^5^ spores/mL. One percent of the inoculum from each culture was then transferred to the corresponding conditions to observe the growth of the indicated strains.

### 2.4. Preparation of Radish Paocai

#### 2.4.1. Stater Culture Preparation

Experimental and reference LAB strains were inoculated into standard strains in MRS broth and incubated at 37 °C for 24 h. After incubation, the LAB cultures were serially diluted and adjusted to a final concentration of 10^7^ CFU/mL. One milliliter of each diluted culture was centrifuged at 4 °C for 10 min at 3000 rpm, and the cell pellets were washed three times with physiological saline to remove residual MRS broth. The washed cell pellets were then resuspended in saline to obtain radish paocai starters containing 10^7^ CFU/mL of LAB strains [6].

#### 2.4.2. Radish Samples Production

Radish, ginger, garlic, and chili pepper were rinsed with tap water to remove any surface contaminants. The radish was peeled, trimmed, and cut into 1.5 × 1.5 × 1.5 cm cubes, while the ginger, garlic, and chili pepper were shredded into strips. Anaerobic fermentation jars (500 mL) were sanitized by boiling in water for 3 min, followed by drying. The following ingredients were then added into each sterile jar: 250 g radish, 10 g NaCl, 2 g sucrose, 7.5 g chili pepper, 10 g ginger, 10 g garlic, 1 g anise, 1 g myrcia, 0.8 g Chinese prickly ash, 0.5 g cassia, and 250 mL boiled cooled water. The contents were thoroughly mixed, the prepared LAB starters were inoculated into the experimental groups, and the jars were labeled with the corresponding strain names. The spontaneous fermentation control group was labeled ‘CK’. Triplicate jars were prepared for each group and were fermented at 25 °C for 7 days.

### 2.5. Acidification of Radish Paocai

To monitor the fermentation process, radish brine samples were aseptically collected from each group on days 0, 1, 2, 3, 4, 5, 6, and 7, and the pH and TTA of the brine samples were determined, as before.

### 2.6. HPLC Measurement of Organic Acids

The organic acids in the radish paocai brine samples were analyzed using high-performance liquid chromatography (HPLC) following a modified method of Chen et al. [24]. The HPLC system (Agilent, 1260, Santa Clara, CA, USA) was equipped with an Agilent SB-Aq chromatographic column (5 μm, 4.6 × 250 nm). Prior to injection, the samples were filtered through a 0.22 μm microfiltration membrane. Each sample was analyzed in triplicate. Isocratic elution was performed using methanol and a mixture of 10 mL of acetonitrile and 3.11 g of NaH_2_PO_4_ in 990 mL of UPW, at a flow rate of 0.8 mL/min. The diode array detector was set at 210 nm and the column temperature was maintained at 25 °C.

### 2.7. FAAs in Radish Paocai

The FAAs in the radish paocai samples were analyzed following a modified method of Chen et al. [24]. Two milliliters of radish paocai brine and 2 g of radish were homogenized and lysed with 5 mL of 6 M HCl solution. Next, 1 mL of digestion solution was mixed evenly with 9 mL sodium citrate buffer, and the mixture was filtered through a 0.22 μm microfiltration membrane before analysis. FAAs were quantified using an automated amino acid analyzer (Hiatchi, la8080, Tokyo, Japan) equipped with an ion-exchange resin column (2622, 4.6× 60 nm). Detection was performed using an ultraviolet (UV) detector set to 570 nm.

### 2.8. Analysis of Taste Contour Using E-Tongue

Fermented radish samples were homogenized in UPW at a 1:1 (*w/w*) ratio, diluted twice with UPW, and were centrifuged to obtain 80 mL of clear supernatant for electronic tongue (E-tongue) analysis using an E-tongue system (Insent, SA-402B, Tokyo, Japan). The E-tongue system contains sensors that can detect nine taste parameters, including umami and richness (AAE sensor), saltiness (CT0 sensor), sourness (CA0 sensor), bitterness and aftertaste-B (C00 sensor), astringency and aftertaste-A (AE1 sensor), and sweetness (GL1 sensor). 

### 2.9. Texture Profile Analysis

Six cubes of radish paocai of a similar size were randomly selected for texture analysis using a texture analyzer (FTC, TMS-Touch, Franklin, Massachusetts, USA) equipped with a TA25 probe to determine hardness, chewiness, and springiness, and a TA44 probe to measure crispness. The TPA parameters were set to a 50% compression ratio and a test speed of 50 mm/min when using the TA25 probe. The puncture test settings included a test speed of 50 mm/min, a post-test speed of 50 mm/min, and a puncture height of 5 mm when using the TA44 probe. The texture was determined according to the methodology described by Zhang et al. [14].

### 2.10. Color Analysis

The a* (red/green), b* (yellow/blue), and L* (lightness) values of each paocai radish sample were measured using a colorimeter (Chroma Meter, CR-400, Osaka, Japan). Each sample consisted of five radish paocai cubes. The C^*^ (chromaticity) and H* (hue) values were calculated using Equations (2) and (3), respectively, based on the a*, b*, and L* values; these color parameters were used to quantify and characterize the color attributes of the radish paocai samples.

(2) $$H^{*}=180/\pi \cdot \arctan(b^{*}{/a}^{*})$$

(3) $$C^{*}={\left(a^{*2}+b^{*2}\right)}^{1/2}$$

### 2.11. Volatile Organic Compounds (VOCs) Analyzed Using GC–IMS

The VOCs in the homogenate, consisting of 0.5 mL brine and 0.5 g radish paocai, were determined using a gas chromatography–ion mobility spectrometer (GC–IMS, GAS, Flavoursec, Italy) equipped with a strong polarity chromatographic column (MXT-WAX, 30 m–0.53 mm, 1 μm). The GC column temperature was maintained at 60 °C during analysis. The carrier gas was high-purity N_2_ (≥99.999%). The carrier gas program was set to a 30 min run time with the following flow rate: 2 mL/min for 2 min, 10 mL/min for 8 min, and 100 mL/min for 20 min. Next, headspace gas sampling was conducted by injecting headspace volatiles (0.5 mL) into the gas chromatograph using a gas-tight syringe. The carrier gas flow rate was set to 150 mL/min and the GC inlet temperature was maintained at 65°C. Cyclohexanone C4–C9 was used as the external standard to determine the retention indices (RIs) of the volatile compounds. By matching the retention times (Rts) and RI values to those of authenticated standards in the database, the tentative identification and semi-quantification of the VOCs present in the samples were achieved [25]. Fingerprint patterns were obtained using the software that came with the instrument.

### 2.12. Sensory Evaluation and Acceptability

Utilizing the training and sensory evaluation methods established by Bao et al. [26], along with relevant Chinese national standards [27,28,29,30], a comprehensive training and assessment protocol was developed in this study. The sensory panel comprised 15 trained faculty members and students from the East China Sea Fisheries Research Institute. In accordance with Andersen et al. [31], four sensory modalities were evaluated to determine the overall acceptability of radish paocai, namely appearance, aroma, texture, and taste. Each attribute was scored on a scale of 0–10, with the total sensory score ranging from 0 to 40. This allowed for the thorough and holistic sensory profiling of paocai samples, in alignment with consumer preferences.

### 2.13. A Comprehensive Evaluation of the LAB Strains Quantified Using TOPSIS

The key physicochemical and sensory indicators affecting radish paocai quality were determined using orthogonal partial least squares discriminant analysis (OPLS-DA), by examining variable importance in projection (VIP) scores. Pearson’s correlation analysis was subsequently conducted between the important indicators (VIP > 1) and sensory evaluation results to elucidate the positive or negative correlational effects on radish paocai quality [26]. Based on the LAB growth kinetics and fermentation performance in radish paocai, the Technique for Order Preference by Similarity to Ideal Solution (TOPSIS) was used to evaluate and rank the LAB strains. The TOPSIS approach establishes hypothetical positive (C^+^) and negative (C^−^) ideal solutions corresponding to the maximum and minimum attribute values across all samples. The relative proximities of the LAB strains to the ideal solutions were calculated to determine the proximities of the LAB strains to the ideal solutions and then to determine the preference order [32]. Generally, the TOPSIS ranking technique comprises four key steps. The first step involves pre-processing the indicator values. After converting the minimum indicators into the maximum indicators (Equation (4)), the evaluation matrix R (R = (b_ij_)_n×m_) is obtained using vector normalization, according to Equation (5).

(4) $$x_{j}^{\prime }=\frac{1}{x_{j}}$$

(5) $$a_{\mathrm{ij}}^{*}=1-\frac{a_{\mathrm{ij}}}{\sqrt{\sum_{i=1}^{n}\;a_{ij}^{2}}},i=1,2,\ldots ,n1\leq j\leq m$$

The second step determines the C^+^/C^−^ ratio for each indicator based on Equations (6) and (7), respectively.

(6) $$c_{j}^{+}=\underset{1\leq i\leq n}{\max}b_{\mathrm{ij}},j=1,\;2,\ldots ,m$$

(7) $$c_{j}^{-}=\underset{1\leq i\leq n}{\max}b_{\mathrm{ij}},j=1,2,\ldots ,m$$

The third step calculates the separation measures representing the distance of each evaluation object from C^+^/C^−^ using Equations (8) and (9), respectively.

(8) $$s_{i}^{+}=\sqrt{\sum_{j=1}^{m}{\left(b_{ij}-c_{j}^{+}\right)}^{2}},\;i=1,2,\ldots ,n$$

(9) $$s_{i}^{-}=\sqrt{\sum_{j=1}^{m}{\left(b_{ij}-c_{j}^{-}\right)}^{2}},\;i=1,2,\ldots ,n$$

Finally, in the fourth step, the relative proximity of each sample to the ideal solution is computed using Equation (10) to generate ranking alternatives from most to least preferred.

(10) $$f_{i}=s_{i}^{-}/\left(s_{i}^{-}+s_{i}^{+}\right),\;i=1,2,\ldots ,n$$

### 2.14. Data Analysis

Statistical analyses of the experimental data, including Pearson’s correlation coefficients, one-way ANOVA, and Duncan’s multiple range test, were performed using IBM SPSS Statistics 25.0. Results are expressed as the mean ± standard deviation (SD), with a confidence interval of *p* < 0.05 indicating statistical significance. Nonlinear regression fitting of the Gompertz model and graphical visualization were performed using Origin 2021. OPLS-DA and associated plotting were conducted in Simca 14.1.0. Graphing of the correlation analysis was conducted in Cytoscape 3.9.1. Comprehensive TOPSIS evaluation calculations were performed using Python 3.10.

## 3. Results and Discussion

### 3.1. Growth Characteristics of the LAB Strains

**Figure 1:**
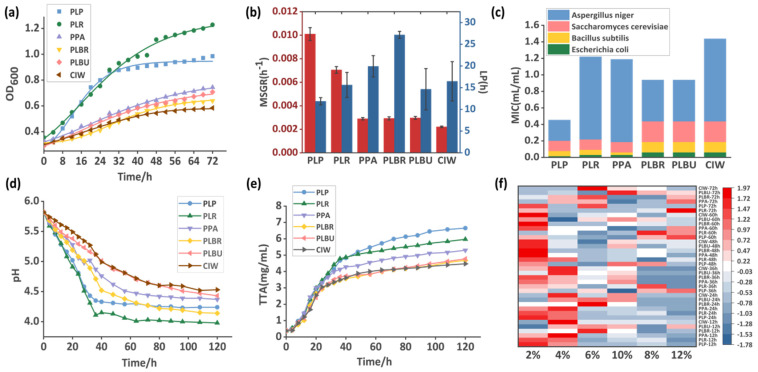
Growth characteristics of the LAB strains. (**a**) Fitting results of the growth curve and the Gompertz model. (**b**) The MSGR and LP of the LAB strains. (**c**) MIC determination results of the fermentation broth supernatants. (**d**) Dynamic changes of the pH. (**e**) Dynamic changes of the TTA. (**f**) Heat map of the growth rates of LAB strains in culture medium with different NaCl concentrations (normalized between groups). Abbreviations: LAB, lactic acid bacteria; MSGR, maximum specific growth rate; LP, lag phase; TTA, total titratable acidity; PLP, *Lactiplantibacillus plantarum*; PLR, *Lacticaseibacillus rhamnosus*; PPA, *Pediococcus acidilactici*; PLBR, *Levilactobacillus brevis*; PLBU, *Lentilactobacillus buchneri*; CIW, *Weissella paramesenteroides*.

The growth kinetics of the LAB strains were modeled using the Gompertz equation. Successful curve fitting was achieved for all strains (R^2^ > 0.99) to determine the maximum specific growth rate (MSGR) and lag phase (LP) parameters (Figure 1a,b). The inoculation of radish juice medium with PLP resulted in the rapid utilization of substrates for growth and metabolism, as evidenced by this strain exhibiting the fastest MSGR and shortest LP, aligning with the pH and TTA measurements (Figure 1d,e). The pH of each fermentation broth rapidly decreased during the initial stages, with PLP and PLR displaying the greatest decline rates, stabilizing after 40 h, whereas the other groups plateaued at approximately 60 h. TTA accumulation showed an inverse correlation with pH, with a rapid production within the first 40 h and declining acidification rates thereafter. At the end of the cultivation period, PLP had the highest TTA, exceeding 6 mg/mL.

The high-salinity environment of paocai alkalizes the microbial profile, which not only affects the selection of starter cultures, but also necessitates NaCl tolerance in potential LAB strains [9]. To evaluate NaCl tolerance, the growth of the LAB strains was monitored across a range of NaCl concentrations (Figure 1f). All strains exhibited a favorable growth and steady increases in OD_600_ at 2% and 4% NaCl; at these concentrations, their high growth rates were maintained. At 6% and 8% NaCl, the growth rates became significantly reduced; however, a measurable growth was still observed. Above 8% NaCl, most strains displayed severely inhibited growth except for PLP and PLBU, which showed excellent tolerance with up to 10% NaCl. The impaired growth of most strains above 6–8% salt concentrations was confirmed by Zhao et al. [4]. The superior NaCl tolerance of PLP and PLBU highlights their potential as starter candidates that can thrive in a high-salinity paocai fermentation environment. 

During fermentation, LAB strains metabolize substances such as acids and bacteriocins to exhibit a potent antibacterial activity against various microorganisms, including spoilage bacteria and fungi [33], especially foodborne microorganisms [8]. In this study, the cell-free supernatants of all LAB fermentations displayed excellent inhibitory effects on *E. coli* at 0.0156–0.0625 mL/mL, with PLP exhibiting the strongest anti-coliform activity (Figure 1c). These results are consistent with previous reports of *Lpb. plantarum* suppression of *Enterobacteriaceae* in fermented carrots, thereby improving food safety [17]. The strains also showed promising inhibitory effects against *B. subtilis* and *S. cerevisiae*, with MIC values ranging from 0.0625 to 0.1250 mL/mL and 0.0125–0.2500 mL/mL, respectively. However, apart from PLP, the antifungal effects on *A. niger* were limited, with PLR, PLBR, and CIW inhibiting mold growth at the highest supernatant concentrations tested. Among the LAB strains screened, PLP displayed the broadest and most potent antimicrobial activity, based on the suppression of all four indicator microorganisms; this may be closely associated with its high organic acids content. This demonstrates the potential of the screened LAB strains to improve paocai consumption and production safety. The production of organic acids and bacteriocins likely contributes to their strong inhibitory effects. The identification of the bioactive metabolites that mediate antimicrobial activities warrants further investigation. 

### 3.2. Dynamic Measurement of Acidification in Radish Paocai

**Figure 2:**
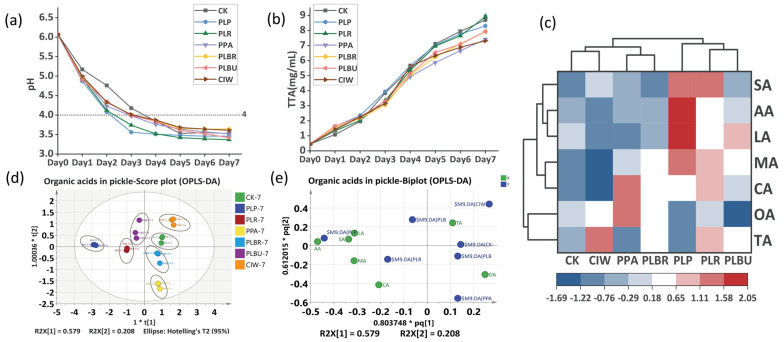
Acidification of radish paocai. (**a**) pH changes in radish paocai. (**b**) TTA changes in radish paocai. (**c**) Clustering heat map of organic acids content (normalized between groups). (**d**) Score plot for the OPLS−DA model. (**e**) Biplot for the OPLS−DA model. Abbreviations: TTA, total titratable acidity; OPLS−DA, orthogonal partial least squares discriminant analysis; PLP, *Lactiplantibacillus plantarum*; PLR, *Lacticaseibacillus rhamnosus*; PPA, *Pediococcus acidilactici*; PLBR, *Levilactobacillus brevis*; PLBU, *Lentilactobacillus buchneri*; CIW, *Weissella paramesenteroides*; MA, malic acid; AA, acetic acid; SA, succinic acid; LA, lactic acid; TA, tartaric acid; OA, oxalic acid; CA, citric acid. (“*t[1]” and “*t[2]” represent the scores of the first and second latent variables, respectively. “R2X[1]” and “R2X[2]” indicate the proportion of variance in the X variables explained by the first and second latent variables.The notations “*pq[1]” and “*pq[2]” represent the positions of samples or variables projected onto the first and second predictive components.)

The pH and TTA dynamics during fermentation are shown in Figure 2a,b, respectively. Inoculated samples, especially PLP and PLBU, displayed rapid pH reductions below 5.00, while CK was 5.18 on day 1, concurrent with significantly higher initial TTA levels compared to the controls (*p* < 0.05). This rapid acidification continued through days 2–3, with the inoculated samples reaching terminal pH values of approximately 4.00. PLP and PLR exhibited the lowest terminal pH values, of 3.56 and 3.54, respectively, when the paocai reached edible acidity by day 3. The uninoculated control initially showed slower pH reductions, with differences between the inoculated and control samples narrowing by day 5 as all groups stabilized. TTA increased linearly throughout the fermentation process for all samples, although differences between the inoculated and control groups remained significant despite the modest overall changes. By day 7, PLR exhibited the highest final TTA of 8.92 mg/mL, whereas PLP and the controls surpassed 8.00 mg/mL. These results demonstrate that inoculation with LAB strains enhances the rate of acid development, particularly early in fermentation, likely because of the substantially higher initial LAB populations that rapidly dominate and acidify the environment [14,34]. The rapid metabolism of sugars into organic acids by the inoculated strains explains the accelerated decrease in pH and increase in TTA. 

During fermentation, LAB strains utilize substrates to produce an array of organic acids [24]. Compared with the controls, the inoculated samples showed increased organic acids levels (Figure 2c), with PLP displaying the greatest enhancement of 40.77%. The highest individual organic acid concentrations were as follows: malic acid (MA), acetic acid (AA), succinic acid (SA), and lactic acid (LA) in PLP at 3.4297, 3.4646, 5.2896, and 2.3162 mg/mL, respectively; tartaric acid (TA) in CIW at 2.2203 mg/mL; oxalic acid (OA) and citric acid (CA) in PPA at 2.6703 and 2.3162 mg/mL, respectively. MA, CA, LA, and AA impart a natural and mild sourness, with sensorially preferred fruity notes. Conversely, OA, TA, and SA can confer slightly unpleasant bitter and astringent flavors, potentially diminishing consumer acceptability [35,36]. According to Figure 2e, the contents of LA, AA, MA, and CA exhibit significant differences in distinguishing PLP and PLR from the other groups. These differences suggest that PLP and PLR may possess a more mild and pleasant aroma, enhancing their overall flavor profile. The organic acid profiles varied independently among the samples (Figure 2d). CK was compositionally similar to CIW, except for in relation to TA content, whereas PPA resembled PLBR based on comparable AA, SA, and LA levels. PLP, PLR, and PLBU also had parallel organic acids constituents. This demonstrates that inoculation with different LAB strains can uniquely modulate organic acid synthesis to tailor the sensory properties. 

### 3.3. VOCs Identification

**Figure 3:**
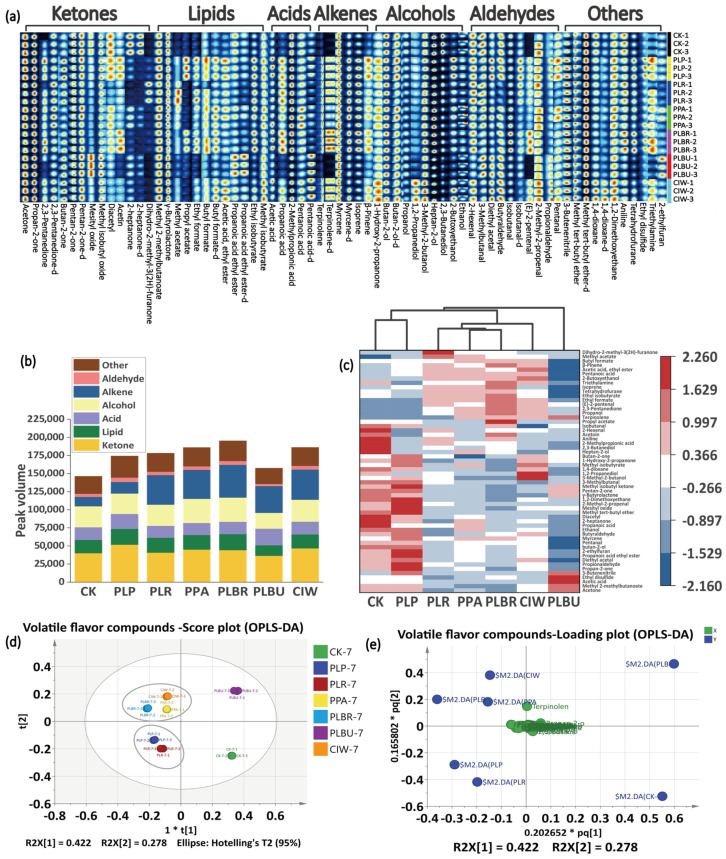
VOC analysis in radish paocai. (**a**) VOC fingerprints in radish paocai as revealed using GC−IMS (−d indicates that the VOC is present in its dimeric form). (**b**) Stack diagram of VOCs based on peak volume. (**c**) Clustering heat map of VOCs (normalized between groups). (**d**) Score plot for the OPLS−DA model. (**e**) Loading plot for the OPLS−DA model. Abbreviations: VOCs, volatile organic compounds; GC−IMS, gas chromatography–ion mobility spectrometer; OPLS−DA, orthogonal partial least squares discriminant analysis; PLP, *Lactiplantibacillus plantarum*; PLR, *Lacticaseibacillus rhamnosus*; PPA, *Pediococcus acidilactici*; PLBR, *Levilactobacillus brevis*; PLBU, *Lentilactobacillus buchneri*; CIW, *Weissella paramesenteroides*. (“*t[1]” and “*t[2]” represent the scores of the first and second latent variables, respectively. “R2X[1]” and “R2X[2]” indicate the proportion of variance in the X variables explained by the first and second latent variables.The notations “*pq[1]” and “*pq[2]” represent the positions of samples or variables projected onto the first and second predictive components.)

GC–IMS detected 90 VOCs after fermentation, with 56 tentatively identified by database matching of retention times and spectral fingerprints (Figure 3a). The overall VOCs profiles and signal intensities showed minimal differences between the spontaneous and inoculated fermentations by endpoint, as visualized in the biplot (Figure 3e). However, certain impact odorants, such as terpinolene, 2-heptanone, dihydro-2-methyl-3(2H)-furanone, methyl acetate, propyl acetate, ethyl formate, and (E)-2-pentenal, contributed to fruity notes and displayed marked concentration variances. These substances were absent or present in relatively low amounts in the CK sample, causing the odor quality of the CK group to be slightly lower than that of the other groups. The substances were categorized into seven classes—ketones, lipids, acids, alcohols, alkenes, aldehydes, and others—to generate a stacked plot based on peak volumes (Figure 3b). PLBU exhibited the highest total VOC content (33.55% higher than that of CK). As a heterotrophic *Lactobacillus brevis* strain, PLBR can utilize diverse substrates to yield more flavorful VOCs, which is in agreement with previous studies [37]. All inoculated samples showed increased VOC levels compared to the controls, especially alkenes and ketones, which are important categories of volatile flavor compounds commonly found in fruits and vegetables. They also constitute the characteristic aromas of many plants. The differences in these two substances between the samples would lead to differences in aroma production, thereby causing differences in sensory quality. CK and PLP displayed comparable VOC profiles, which is consistent with the results of Luo (2023). Cluster analysis grouped CIW, PPA, and PLBR (Group 1), as well as PLP and PLR (Group 2), based on similarities, whereas CK and PLBU were distinct (Figure 3d). Clustering differed slightly from OPLS-DA, likely owing to variations in the calculation method.

### 3.4. Taste Contour and FAAs Analysis

**Figure 4:**
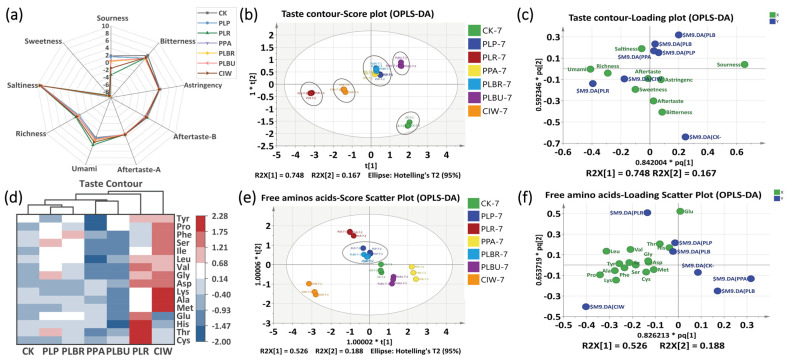
Taste contour and FAAs analysis of radish paocai using the OPLS−DA model. (**a**) Radar chart of the taste contour on the 7th day. (**b**) Score plot of the taste contours. (**c**) Loading plot of the taste contours. (**d**) Clustering heat map of FAAs (normalized between groups). (**e**) Score plot of FAAs. (**f**) Loading plot of FAAs. Abbreviations: FAAs, Free amino acids, OPLS−DA, orthogonal partial least squares discriminant analysis; PLP, *Lactiplantibacillus plantarum*; PLR, *Lacticaseibacillus rhamnosus*; PPA, *Pediococcus acidilactici*; PLBR, *Levilactobacillus brevis*; PLBU, *Lentilactobacillus buchneri*; CIW, *Weissella paramesenteroides*. (“*t[1]” and “*t[2]” represent the scores of the first and second latent variables, respectively. “R2X[1]” and “R2X[2]” indicate the proportion of variance in the X variables explained by the first and second latent variables.The notations “*pq[1]” and “*pq[2]” represent the positions of samples or variables projected onto the first and second predictive components.)

Radar charts depicted the dynamic changes in the taste profiles of the seven samples during fermentation (Figure 4a). Sourness progressively intensified, whereas sweetness, umami, and richness gradually declined; this was attributed to LAB substrate utilization producing organic acids and volatiles [34]. On Day 7, PLR and CIW exhibited the highest richness and umami, CK had the highest bitterness, and CK and PLP had the highest sourness. Sourness and richness constituted the most marked taste differentials, driving sample distinctions (Figure 4c,d). CK differed significantly from the inoculated samples, whereas PLBR, PLP, and PPA displayed the greatest taste profile similarities among the LAB-inoculated fermentations. 

In addition to organic acids, sugars, and inorganic ions, FAAs play crucial roles in taste development by contributing to sweetness, umami, bitterness, and sourness. However, FAAs often elicit multiple taste sensations, adding complexity to taste analysis [38]. The total FAAs in CIW and PLP exceeded 33 mg/g, which was approximately 18% higher than that in CK, demonstrating the feasibility of enhancing the paocai taste with LAB inoculation. Elevated umami levels in PLR and CIW potentially stemmed from higher concentrations of glutamate and aspartate (*p* < 0.05), which are known sources of umami taste (Figure 4d). The relatively high amount of total FAAs in PLR and CIW may also confer a richer taste. FAAs exhibit irregular dynamics during fermentation, as they are converted into intermediary metabolites [39]. The OPLS-DA models showed that CIW was the most distinct in the FAAs profile, whereas PLBR and PLP were the most similar (Figure 4e). PLBU, CK, and PPA clustered farthest from the FAA data points, indicating a poor FAA performance (Figure 4f).

### 3.5. Texture Analysis of Radish Paocai

**Figure 5:**
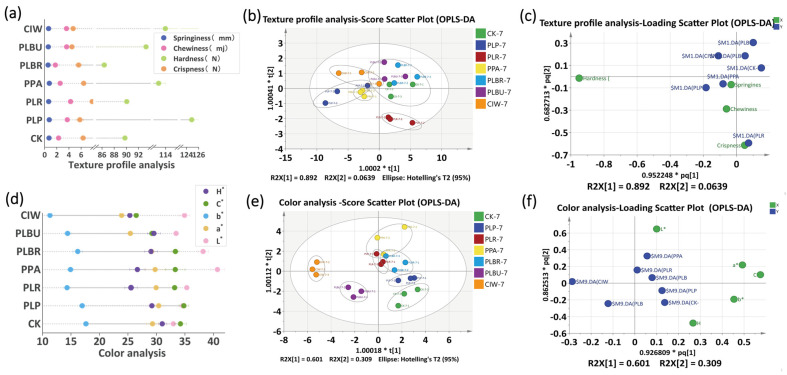
Texture parameter and color analyses of radish paocai. (**a**) Textures on the 7th day. (**b**) Score plot of the textures. (**c**) Loading plot of the textures. (**d**) Color determinations on the 7th day. (**e**) Score plot of the colors. (**f**) Loading plot of the colors. Abbreviations: OPLS−DA, orthogonal partial least squares discriminant analysis; PLP, *Lactiplantibacillus plantarum*; PLR, *Lacticaseibacillus rhamnosus*; PPA, *Pediococcus acidilactici*; PLBR, *Levilactobacillus brevis*; PLBU, *Lentilactobacillus buchneri*; CIW, *Weissella paramesenteroides*. (“*t[1]” and “*t[2]” represent the scores of the first and second latent variables, respectively. “R2X[1]” and “R2X[2]” indicate the proportion of variance in the X variables explained by the first and second latent variables.The notations “*pq[1]” and “*pq[2]” represent the positions of samples or variables projected onto the first and second predictive components.)

Texture is a key quality attribute for the consumer acceptance of paocai, although it softens during fermentation [14]. Pectin and fiber decomposition by microbial pectinases and cellulases likely cause textural changes in radish paocai, as high salt and microbial activity promote pectin and cell wall breakdown [40]. Declining springiness, chewiness, hardness, and crispness supported this notion, with hardness being the most critical texture parameter (Figure 5a,c). After fermentation, PLP displayed the highest hardness, which was 44.58% greater than that of the PLBR. Springiness was uniformly low across all the samples (Figure 5c). Chewiness and crispness varied slightly, with PLR exhibiting optimal traits (*p* < 0.05). In the analysis of texture, except for in the PLR group, the independence of the other sample groups is not satisfactory. This is due to the higher crispness of the PLR group, while the performance of the other samples is relatively close (Figure 5b,c). Although not statistically significant, disparate LAB species differentially affected paocai texture.

### 3.6. Determination of Color Indicators

The radish paocai fermented in this study was *Raphanus sativus* L., containing abundant anthocyanidins with a high water solubility [41]. Uniform red cube coloration arose as anthocyanidins dissolved during fermentation, markedly elevating H*, while slightly decreasing a*. In the early fermentation stage, particularly the first three days, the a* shift in CK was most pronounced. PLP and PLR also exhibited high a* and L* values throughout fermentation, likely attributable to rapid acid generation initially. Organic acids like lactic acid possess antioxidant properties, thus higher organic acid yields with inoculated fermentations early on may have improved color parameters [36]. As microbial metabolism, endogenous enzymes, and oxidation reactions progressed, radishes transitioned to yellow and black hues, increasing b*. At the fermentation endpoint, CK displayed the highest b* and lowest L* (Figure 5d), potentially due to a slower initial pH decrease inadequately preventing enzymatic browning [42]. The greater color deterioration in CK suggests that inoculation with *Lpb. plantarum* or *Lcb. rhamnosus* helps to stabilize anthocyanidins and other pigments during fermentation through rapid acidification and antioxidant effects. However, all samples still experienced some enzymatic browning over time, indicating that additional protective measures may further improve paocai color retention. At the end of fermentation, PLBR and PLR exhibited higher L* values, potentially making the samples not visually appealing to consumers. Hue is typically measured in degrees on the color wheel, where 0° corresponds to red, 120° to green, 240° to blue, and values in between these numbers represent intermediate hues. The results indicate that the color of CIW and PLR kimchi is more reddish because their H* values are closer to 0°. Chroma refers to the purity or intensity of a color. It indicates how strong or weak a color appears. The results show that CIW has the lowest C* value, followed by PLBU, while the other groups have higher and relatively similar C* values. (Figure 5d). According to Figure 5e,f, the samples were mainly categorized into three groups based on color measurements—CIW, PLBU, and the remaining samples. This grouping is primarily due to differences in L* and H* values.

### 3.7. Correlation Analysis of Sensory Evaluation and Important Indicators

**Figure 6:**
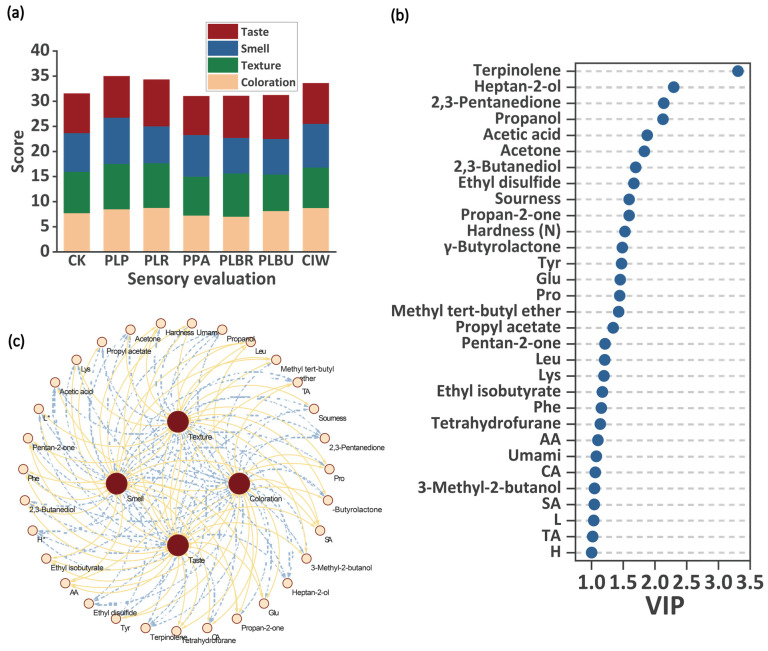
Sensory evaluation and correlation analysis. (**a**) A stacked plot of the sensory evaluation scores. (**b**) Selection of important indicators (VIP > 1). (**c**) Correlation analysis between the sensory evaluation and the important indicators (positive and negative correlations represented by yellow solid and blue dashed lines, respectively). Abbreviations: VIP, variable importance in projection; PLP, *Lactiplantibacillus plantarum*; PLR, *Lacticaseibacillus rhamnosus*; PPA, *Pediococcus acidilactici*; PLBR, *Levilactobacillus brevis*; PLBU, *Lentilactobacillus buchneri*; CIW, *Weissella paramesenteroides*; AA, acetic acid; SA, succinic acid, TA, tartaric acid; CA, citric acid.

Sensory evaluation has become a vital tool in food research for ensuring the consumer acceptance of products [12]. The overall sensory scores showed that PLP had the highest value (Figure 6a). Correlating sensory and instrumental metrics can supplement subjective quality appraisals with numerical data [32]. PLP, PLR, and CIW exhibited excellent taste, with scores exceeding 8.5, aligning with the heightened consumer perception of these attributes during evaluation [31]. Crispness typifies Sichuan paocai. Accordingly, PLP had the highest texture score, corroborating the TPA results. PLR and PLP ranked first in terms of color and flavor. 

Because of the numerous indicators, it is necessary to use appropriate chemometric tools to extract specific indicators with high specificity and response values from complex data. OPLS-DA is an improved method based on PLS-DA, which can eliminate data unrelated to class information through orthogonalization. Consequently, OPLS-DA can more easily exclude irrelevant independent variables and identify characteristic variables of the samples [43]. Variables with a VIP > 1 are generally considered to have greater differences between classes and play a crucial role in classification. This approach has been applied in numerous studies [44]. According to the calculations, there were 31 indicators with a VIP > 1, including 16 indicators of VOCs, four of organic acids, four of taste contours, six of FAAs, one of TPA, and two of color (Figure 6b). Among them, 18 indicators were positively correlated with sensory evaluation, while 13 indicators were negatively correlated with sensory evaluation (Figure 6c). Sensory analysis indicated that inoculation with *Lpb. plantarum* or *Lcb. rhamnosus* enhanced key textural, flavor, and appearance attributes of Sichuan paocai compared to uninoculated controls.

### 3.8. Comprehensive Evaluation of the LAB Strains Using TOPSIS

A single sample can exhibit varied attributes, posing challenges for holistic quality assessment. Selecting an optimal methodology for a comprehensive evaluation is critical [26,32]. TOPSIS offers straightforward and robust integration, as evidenced by its widespread implementation across material science, food science, and mechanical engineering [45]. 

In total, 18 positively and 14 negatively correlated metrics were designated as maximum and minimum indicators, respectively. MRSH was the maximum indicator based on favorable LAB growth kinetics, whereas MIC and MH were minimal. By unifying all the generated data, the matrix R was obtained (Equation (11)). Calculating C^+^/C^−^ distances using Equations (14) and (15) enabled the ranking of the strains by their relative closeness to the ideal solution F, as follows: f_PLP_ > f_PLR_ > f_PLBR_ > f_CIW_ > f_PPA_ > f_PLBU_. PLP, PLR, and PLBR showed superior capacities, with higher comprehensive scores than the standard CIW. TOPSIS facilitates the effective ranking of complex fermentation attributes to identify optimal starter cultures. This technique comprehensively integrates diverse biochemical and microbiological data that are otherwise difficult to simultaneously compare. Future studies could apply TOPSIS to additional LAB species and fermentation conditions to further validate and refine culture selection.

(11) $$R=\left[\begin{array}{ccccc} 0.4006 & 0.4761 & \ldots & 0.8278 & 0.7349\\ 0.5415 & 0.3928 & \ldots & 0.5369 & 0.6509\\ 0.4002 & 0.4023 & \ldots & 0.5487 & 0.5552\\ 0.4802 & 0.3736 & \ldots & 0.6438 & 0.3935\\ 0.2299 & 0.3717 & \ldots & 0.6438 & 0.6730\\ 0.3205 & 0.4237 & \ldots & 0.4538 & 0.6323 \end{array}\right]$$

(12) $$C^{+}=\left[0.5415,\;0.4761,\ldots ,0.8278,\;0.7349\right]$$

(13) $$C^{-}=\left[0.2299,\;0.3717,\ldots ,0.4538,\;0.3935\right]$$

(14) $$s^{+}=\left[0.9751,\;0.9999,\;1.3818,\;1.0960,\;1.4636,\;1.2507\right]$$

(15) $$s^{-}=\left[1.1891,\;1.1949,\;0.6768,\;1.1372,\;0.5703,\;1.0108\right]$$

(16) $$F=\left[f_{\mathrm{PLP}},f_{\mathrm{PLR}},f_{\mathrm{PPA}},f_{\mathrm{PLBR}},f_{\mathrm{PLBU}},f_{\mathrm{CIW}}\right]=\left[0.5495,\;0.5444,\;0.3288,\;0.5092,\;0.2804,\;0.4469\right]$$

## 4. Conclusions

This study comprehensively evaluated the fermentative capacities and flavor effects of five LAB strains on radish paocai using TOPSIS ranking. The results demonstrated that all strains exhibited robust growth on radish substrates, good osmotolerance, and antimicrobial activity, enabling adaptation to high-salt paocai brine and enhanced safety. Inoculated fermentation produced acids rapidly, shortening fermentation by two days versus spontaneous fermentation. Inoculation also increased organic acids, volatile compounds, and free amino acids to an extent, thereby improving flavor and taste. Accelerated acidification likely slows color deterioration and enhances pigment stability. The inoculated samples exhibited excellent textural enhancement. The TOPSIS ranking identified *Lpb. plantarum*, *Lcb. rhamnosus*, and *Lvb. brevis* as the top performers with higher scores than the CIW starter. These could serve as alternative cultures to improve the existing paocai inocula. Overall, this study provides new data on LAB characteristics and quality impacts to advance paocai starter research. These findings demonstrate that targeted strain selection can be used to tailor biochemical transformations for desired sensory and functional properties. Due to the varying performances of different strains in terms of the fermentation characteristics of paocai, future studies could explore the possibility of synergistic fermentation among multiple strains, aiming to achieve better outcomes than single-strain inoculation.

## Data Availability

The original contributions presented in the study are included in the article, further inquiries can be directed to the correspondings authors.
